# The need for effective interprofessional collaboration between nutrition and dentistry

**DOI:** 10.3389/fpubh.2025.1534525

**Published:** 2025-02-26

**Authors:** Jillian Kaye, Sara Lee, Courtney H. Chinn

**Affiliations:** College of Dentistry, Department of Pediatric Dentistry, New York University, New York, NY, United States

**Keywords:** nutrition, oral health, interprofessional education, collaborative care practice, education, registered dietitian nutritionist

## Abstract

There are bidirectional relationships between diet and nutrition, systemic health and oral health. Diet and nutrition are fundamental to the prevention and management of chronic diseases. Systemic health can impact oral health. And oral health can influence diet and nutrition. The 2020 Surgeon General’s Report “Oral Health in America” stated that nutrition is an integral factor in the development of oral disease and health overall. Within the medical model, Registered Dietitians have been impactful members of a multidisciplinary team by providing medical nutrition therapy and nutritional counseling to reduce the development and management of chronic diseases like obesity, diabetes and cardiovascular disease. Despite the well-established collaboration and the role nutrition plays in health outcomes, there is limited documented collaboration between nutrition and dentistry. The purpose of this paper is to review the current evidence of nutritional counseling in oral health settings compared to the medical model, identify specific challenges, and suggest possible next steps for collaboration. The intended outcome of this paper will be to provide the reader with insight about the need to integrate nutrition into both oral health education and clinical practice to address prevalent chronic diseases and increase health equity for those at high risk.

## Introduction

Nutrition plays a vital role in the bidirectional relationship between oral and systemic health. Diets high in refined carbohydrates, particularly refined sugars, and acidic foods can increase the risk for both obesity and dental caries. Oral health can impact prevention and management of chronic diseases and the presence of chronic diseases can present as symptoms in the mouth. Patients with poorly controlled diabetes are three times more likely to develop periodontal disease than non-diabetic patients (1). This is due to numerous factors including physiological changes in blood flow to the gums, systemic and localized inflammation, as well as insulin resistance and xerostomia (2). Conversely, periodontal disease can also inhibit a diabetic patient’s ability to manage their blood glucose levels increasing their susceptibility for infection, systemic inflammation and poor wound healing (3). The nutritional aspects of a diet and dietary behaviors can directly impact the progression of oral disease while oral disease and lack of dentition can impact the ability to meet nutritional needs. Individuals with nutrient poor diets, low in fruits and vegetables, are at an increased risk of developing vitamin and mineral deficiencies which can present in the mouth as gingival bleeding, angular cheilitis or stomatitis (4, 5). Similarly, the lack of teeth and oral pain can impact an individual’s ability to eat, particularly nutrient-dense foods such as proteins, fruits, and vegetables, potentially leading to vitamin and mineral deficiencies (6).

Oral diseases such as dental caries and periodontal disease affects individuals across the lifespan. Dental caries is the most common childhood chronic disease, five times more common than asthma (7). In the United States (US)nearly 46% of children experiencing untreated or restored dental caries in primary or permanent dentition (8). As a result, emergency care for dental caries accounts for an average of 34 million hours of missed school annually (9). Furthermore, more than one in five adults have an untreated cavity and nearly 40% deal with some level of periodontal disease (10). As older adults retain their teeth longer throughout their life, 96% of individuals over the age of 65 have had one cavity in their life and one in six currently have an untreated cavity (5). If left untreated, cavitated teeth in adults and older adults can lead to loss of functional dentition (11). Disruptions to mastication can inhibit normal dietary intake, increasing the risk for nutrition-related consequences like vitamin-mineral deficiencies and malnutrition (12). These statistics emphasize the critical importance of oral health across all age groups and how the prevention and management of oral disease can significantly impact overall health.

Despite this widely understood role of nutrition in the bidirectional relationship between oral and systemic health and the global impact of oral disease, dental professionals have remained relatively isolated from other health care providers, most notably with nutrition professionals like Registered Dietitian Nutritionists (RDN). The role of a RDN as part of the medical team has been highlighted in the prevention and management of chronic diseases like cardiovascular disease, obesity, diabetes, and kidney disease (13–17); however, there are limited documented collaborations between dental and nutrition professionals both in the training and clinical practice. This mini review will provide perspective on the US’s model for interdisciplinary collaborations between RDNs in the medical field, compare and explore current integrations of nutrition within the dental field and discuss solutions for challenges facing continued progress between nutrition and oral health professionals.

## Nutrition education in medicine

Within the medical model, diet and nutrition has been recognized to play a significant role in the prevention and management of chronic diseases (8). Dietary modifications have been shown to be effective management techniques when discussed by a trained medical team and implemented with a patient (18). To ensure this type of interprofessional care can meet a patient’s individual needs, it is essential to cultivate a trained workforce for collaboration through interprofessional education. Nutrition has been an integral part of medical education in the United States (US) since the mid-20^th^ century. In 1963, the American Medical Association (AMA) Council on Food and Nutrition was the first to note the urgent need for medical schools to “define responsibilities and challenges” in teaching nutrition (19). This declaration was critical in encouraging US medical schools to develop and integrate formal nutrition education into both medical curricula and professional practice. Around the same time, the outcomes from the first White House Conference on Food, Nutrition, and Health in 1969 similarly stated that nutrition was inadequate in medical education and advocated for funding to be available for future program development. Throughout the 20^th^ century, the emphasis on nutrition education in medical schools varied leading to inconsistent implementation nationwide. In 1985, the National Academy of Science recommended that medical schools provide a minimum of 25 h of nutrition education to equip graduates with the ability to perform basic nutrition assessments, counsel patients on prevention and treatment of chronic diseases, and understand when to refer to a RDN (20). Despite all efforts, a recent study found that 71% of medical schools are not currently meeting these recommendations. As a result, physicians report feeling unprepared to approach these critical aspects of patient care and agree that additional training in nutrition would allow them to provide better clinical care (21).

## The registered dietitian nutritionist as part of the medical team

Despite physicians’ lack of confidence in providing nutrition education themselves, they are taught the significance of referring patients to RDNs. RDNs have been part of interprofessional care within the medical model for years and demonstrate how effective interprofessional collaborative care results in a safe, comprehensive, coordinated health care system with improved patient health outcomes (22). As a result of integrating nutrition into medical education, the role of a RDN’s participation as a key member of the medical health care team is now widely established. RDNs commonly work in hospitals, private practice, or other health care facilities as part of a multidisciplinary team. RDNs have significantly impacted health outcomes through medical nutrition therapy (MNT), a form of treatment that uses nutrition education and behavioral counseling to prevent or manage chronic diseases. MNT enables RDNs to offer personalized, evidence-based nutrition recommendations for managing chronic diseases such as cardiovascular disease, obesity, diabetes, and kidney disease (13–17) while improving overall health. For instance, RDNs are employed to ensure patients are receiving adequate nutrition in and out of the hospital. Physicians often refer high-risk patients to RDNs to monitor their nutritional intake, verifying appropriate diets and supplements are ordered for timely discharge. While MNT can be provided to all patients, insurances require specific diagnosis and documentation for coverage, which restricts access for many patients. Due to the disparity in nutrition preparedness and lack of insurance coverage, most medical professionals feel more comfortable referring to a nutrition professional instead of providing nutrition education themselves. This has led to a prolonged and continued siloing of medical and nutrition professionals, outside of traditional in-patient settings. However, despite these limitations, the medical field’s integration of nutrition can be used as a model for the progression of nutrition into dentistry.

## Nutrition education in dentistry

The paradigm shift in dentistry to health promotion and disease prevention has acknowledged nutrition as a noninvasive bridge between oral and systemic health (23). With the medical model as an example, nutrition can be an integral part of training for dental providers with adequate requirements and appropriate funding. The 2020 Surgeon General’s report on “Oral Health in America” supports interprofessional education by affirming that oral health is more than just healthy teeth. It highlights that diet and nutrition are major multifactorial environmental factors in the development of oral disease (5). Understanding the distinct roles and responsibilities of RDNs and dental professionals can encourage collaborative efforts to address how dietary choices influence oral health. For instance, the American Dental Association (ADA) (24), American Dental Hygienists Association (ADHA) (25) and the American Academy of Pediatric Dentistry (AAPD) (26) have issued nutrition guidelines for dental practitioners. Similarly, the Academy of Nutrition and Dietetics (AND) has released a position statement emphasizing the importance of nutrition and diet in oral health (27). In spite of this universal understanding of the link between diet and oral disease risk, dental education has yet to make nutrition a key component of training for dental professionals.

Compared to physicians who primarily see patients with illnesses, dentists see patients regularly for health maintenance providing a unique opportunity to integrate lifestyle management, such as diet, as a component of oral hygiene education (28). The ADA’s Commission on Dental Accreditation (CODA) sets the standards for dental education across the US. CODA has reviewed and approved nutrition education topics for both didactic and clinical components of predoctoral dental education. While there is no standardized requirement or optimal number of hours for nutrition education (28), an average 16 h of didactic education was dedicated to nutrition (29). This number has remained constant and relatively small in comparison to other topics. Comprehensive nutrition education is seldomly presented independently and addressed in conjunction with other preclinical courses in the early years of dental school. Individual dental schools have taken their own initiatives to incorporate nutrition into the training through the use of clinical nutrition case seminars (28) and an online food and nutrition module (30). These advancements are intended to help dental students feel confident in providing nutrition counseling within their scope of practice (27).

Dental education spends more time on clinical nutrition integration compared to classroom instruction (28, 29), with a majority of dental schools reporting that their students assess nutrition risk through a standard caries risk assessment tool as part of routine dental care (28, 29). CODA states that dental schools should assess students nutritional competency by providing their trainees “in-depth understanding of basic biological principles, consisting of a core of information on fundamental structures, function and interrelationships of the body.” Dental students may be asked to assess their patient’s nutrition status clinically, but may not have been provided with the foundational education for effective nutrition counseling. While assessing a patient’s nutrition status is repeated through caries risk assessment tools clinically, nutrition counseling topics are rarely reassessed, limiting reinforcement for dental students to feel comfortable with this skill set. Presenting nutrition topics to dental students throughout their 4 years of training can increase the chance that nutrition education will be embraced into their care (28).

At the same time, nutrition topics are generally taught by a dental faculty with a background in general science or a biochemist from “nutrition-related disciplines” (23, 28). The need for dental professionals to collaborate with RDNs to facilitate referrals have been identified but practices have not been documented (27). Across the US, less than 10 dental schools employ RDNs with varying levels of integration into patient care. As a result, dental students may receive limited training on how to work collaboratively with nutrition professionals in a clinical setting (29). Additionally, RDNs may be unaware of their role in management of oral disease and unprepared to work alongside dental professionals. Most dietetic internships do not adequately provide dietetic interns the proper training to seek this type of employment or feel qualified to teach dental related topics.

## The need for a registered dietitian nutritionist as part of the dental team

Similar to the minimal presence of RDNs in dental academia, there is a lack of representation of RDNs in dental practice. Dental practices hardly have employed a RDN, with few going as far as having staff members with a nutrition background (31). This lack of collaborative care practices between nutrition and dental professionals impacts a patient’s ability to receive comprehensive dental care. RDNs are the only trained professionals to provide MNT, however this does not extend to dental caries. A patient’s medical insurance status can deter them from receiving MNT, as it requires specific diagnosis and documentation for coverage. However, insurance limitations are not exclusive to medicine. In dentistry, procedures and dental diagnoses are documented using the Current Dental Terminology (CDT). The only nutrition-related code is D1310, “nutritional counseling to help control dental disease.” Reimbursement for this code depends on the patient’s need, the state of service and the dental insurance plan. Submitting a claim requires a comprehensive dental exam code and note which includes a caries risk assessment, dietary habits, and specific dietary-related recommendations. If dental professionals do not allot time for oral health-focused nutrition counseling, they may be unable to receive reimbursement, which could deter them from providing these services at all.

## Discussion

The opportunity for effective collaboration between dental and nutrition professionals is achievable, but the factors limiting this type of multidisciplinary team must be addressed. It starts with training an adequate workforce. Dietetic internships should provide ample time in the curriculum to allow interns to learn about the role of nutrition in the progression of oral disease, as well as offering specific rotations in oral health settings. This incorporation would provide the nutrition workforce an understanding of their role in collaboration and prepare them to seek employment to educate and work alongside oral health professionals. The connection between nutrition, oral and overall health is widely understood, but enhancing this type of collaboration can lead to specific MNT recommendations at preventing and managing oral disease.

Dental schools should consider having nutrition-oral health related topics taught by nutrition professionals. The accreditation board would make nutrition a fundamental part of dental curricula with a set number of hours and topics to be covered. This would not only provide dental trainees evidence-based education and up-to-date recommendations, but it would also foster interdisciplinary education and care. This would open up opportunities for employment for RDNs in dental education as well.

Expanding insurance coverage for nutrition counseling in a dental setting should be considered. Having RDNs on staff would allow expansion of caries risk assessment tools to encompass both caries and nutrition risk. Moreover, having a credentialed nutrition professional would provide an opportunity for patients to receive MNT, nutrition-focused counseling outside the scope of practice of a dentist. The CDT code D1310 has limited reimbursement rates leading dental providers to rarely submit this code. Limited use of this code has reduced an understanding of the impact of nutrition counseling from dental professionals on their patient’s dietary behaviors, compared to MNT, which has been shown to be beneficial in prevention and management of chronic disease (32). However, providing MNT involves a complexity of insurance and reimbursement issues (33). A recent bill has been proposed to expand coverage for MNT to include additional diseases such as obesity and unintended weight loss in older adults—conditions that interplay with oral disease (34). Although this new bill does not explicitly cover oral disease or include dental insurance codes, it signals the opportunity for future coverage due to the established connection between nutrition and oral health. Lastly, significant changes in both education and insurance coverage in the medical model occurred when major organizations such as the White House endeavored to advance food and nutrition initiatives. For the first time in 50 years, the White House hosted a conference on Hunger, Nutrition and Health in 2022, focusing on improving access to affordable food and educating health care providers (35).

With the paradigm shift in dentistry and both local and systemic resources focusing on enhancing nutrition education and access, this is an optimal time for such collaboration. Ensuring collaboration between dental and nutrition professionals will not only benefit the training of future healthcare providers but will also have an impact on all aspects of oral health patient care.
